# Low-dose 7,8-Dihydroxyflavone Administration After Status Epilepticus Prevents Epilepsy Development

**DOI:** 10.1007/s13311-022-01299-4

**Published:** 2022-09-30

**Authors:** Annunziata Guarino, Barbara Bettegazzi, Nimra Aziz, Mario Barbieri, Daniela Bochicchio, Lucia Crippa, Pietro Marino, Maddalena Sguizzato, Marie Soukupova, Silvia Zucchini, Michele Simonato

**Affiliations:** 1 Department of Neuroscience and Rehabilitation, University of Ferrara, via Fossato di Mortara 70, 44121 Ferrara, Italy; 2 University Vita-Salute San Raffaele, via Olgettina 58, 20132 Milan, Italy; 3 Laboratory of Technologies for Advanced Therapy (LTTA), Technopole of Ferrara, Ferrara, Italy; 4 Division of Neuroscience, IRCCS San Raffaele Hospital, via Olgettina 60, 20132 Milan, Italy

**Keywords:** Brain-derived neurotrophic factor, TrkB receptor, Epileptogenesis, Neuronal death

## Abstract

Temporal lobe epilepsy often manifests months or even years after an initial epileptogenic insult (e.g., stroke, trauma, status epilepticus) and, therefore, may be preventable. However, no such preventive treatment is currently available. Aim of this study was to test an antioxidant agent, 7,8-dihydroxyflavone (7,8-DHF), that is well tolerated and effective in preclinical models of many neurological disorders, as an anti-epileptogenic drug. However, 7,8-DHF also acts as a TrkB receptor agonist and, based on the literature, this effect may imply an anti- or a pro-epileptogenic effect. We found that low- (5 mg/kg), but not high-dose 7,8-DHF (10 mg/kg) can exert strong anti-epileptogenic effects in the lithium-pilocarpine model (i.e., highly significant reduction in the frequency of spontaneous seizures and in the time to first seizure after status epilepticus). The mechanism of these different dose-related effects remains to be elucidated. Nonetheless, considering its excellent safety profile and antioxidant properties, as well as its putative effects on TrkB receptors, 7,8-DHF represents an interesting template for the development of effective and well-tolerated anti-epileptogenic drugs.

**Supplementary Information:**

The online version contains supplementary material available at 10.1007/s13311-022-01299-4.

## Introduction

Some forms of epilepsy, for example, mesial temporal lobe epilepsy (mTLE), can originate months or even years after a brain-damaging event, e.g., injury, stoke, status epilepticus (SE), infection [1]. While patients can recover from this initial event, it may set in motion a series of alterations at molecular, cellular, and circuitry level that, in time, lead to the transformation of a normal brain into epileptic, i.e., to the appearance of spontaneous seizures. This process is called epileptogenesis. In principle, these forms of epilepsy should be preventable. In practice, this is extremely difficult, because only a subset of the individuals who experience an epileptogenic insult will later become epileptic, and no reliable biomarker is currently available to predict who will and who will not [1]. This implies the practical impossibility of clinically testing potential preventive therapies [2, 3].

The question posed by the epileptogenesis process can be viewed as an example of a general neuroscience question: how a fleeting event (a life experience in general, an epileptogenic event in our case) can lead to permanent changes in the structure and function of the brain. One hypothesis may be the accumulation in the lesion area of endogenous molecules that can be responsible for the structural and functional alterations mentioned above. Such molecules should therefore [1] increase after the epileptogenic insult and [2] have a profile of actions coherent with an involvement in neural circuitry plastic changes ultimately resulting in hyperexcitability [4]. One molecule that meets these criteria is the brain-derived neurotrophic factor (BDNF).

Epileptogenic insults increase BDNF signaling. For example, SE increases BDNF expression [5–7] and causes enhanced activation of the BDNF high-affinity receptor, TrkB [8, 9]. Less clear is whether TrkB hyper-activation plays a pro- or anti-epileptogenic role. A local supplementation of BDNF together with another neurotrophic factor, fibroblast growth factor-2 (FGF-2), has been reported to attenuate SE-induced cell damage, increase hippocampal neural stem cell proliferation and neuronal differentiation, and reduce the aberrant aspects of epileptogenesis-associated neurogenesis, thereby ameliorating the epilepsy pathology and reducing the frequency and severity of spontaneous seizures [10]. In addition, a partial agonist of the TrkB receptor (LM22A-4) has been reported to enhance structural and functional measures of GABAergic inhibition and to suppress post-traumatic epileptogenesis when administered after cortical injury [11].

However, other lines of evidence suggest that BDNF is instead pro-epileptogenic: transgenic overexpression of BDNF is sufficient to cause mTLE in adult mice [12, 13], and BDNF heterozygotes exhibit impairments in kindling epileptogenesis [14], an effect observed also after intraventricular administration of proteins that selectively scavenge BDNF [15]. The most compelling evidence of a pro-epileptogenic role of BDNF, however, comes from transgenic mice carrying a genetic modification in the TrkB kinase domain (TrkB^F616A^) that renders the receptor sensitive to inhibition by an otherwise inert blood–brain barrier-permeable small molecule, 1NMPP1. Treatment with 1NMPP1 for 2 weeks after intra-amygdala kainate (KA)-induced SE prevents development of mTLE and comorbid anxiety-like behavior in TrkB^F616A^ transgenic animals [16]. However, this also exacerbates SE-induced neuronal degeneration [17]. In brief, converging evidence supports a neuroprotective effect of BDNF, but data are conflicting on its effect on epileptogenesis.

Aim of the present study was to test a promising BDNF agonist, 7,8-dihydroxyflavone (7,8-DHF), that proved to be well tolerated and effective in preclinical models of many neurological disorders [18]. In addition to its effect on TrkB, this compound is known to exert antioxidant effects [19]. Because reactive oxygen species are rapidly induced in the brain after epileptogenic insults, and antioxidant drugs have been reported to exert anti-epileptogenic effects [20], 7,8-DHF is expected to exert favorable effects. However, its actions on BDNF may represent a double-edged sword.

## Methods

### Animals

All experiments were performed in male Sprague–Dawley rats (Envigo, Udine, Italy) weighing 200–250 g. Animals were kept under standard housing conditions: room temperature 22–24 °C, 12-h light/dark cycle, and free access to food and drinking water. They were allowed to adapt to laboratory conditions for at least 1 week before starting the experiments. All experimental protocols were approved by the University of Ferrara Committee for Animal Welfare and by the Italian Ministry of Health (D.M. 90/2021-PR) and were carried out in accordance with the guidelines of the National Institute of Health and the European Community (EU Directive 2010/63/EU) on the Use and Care of Animals. In addition, all experimental procedures have been performed following the ARRIVE (Animal Research: Reporting in Vivo Experiments) and the NC3Rs (National Centre for the Replacement, Refinement and Reduction of Animal Research) guidelines [21, 22].

### Lithium-Pilocarpine Model

Rats were administered 127 mg/kg lithium chloride by gastric gavage. After approximately 14 h, they received a subcutaneous injection of methyl-scopolamine (1 mg/kg, Sigma-Aldrich, Saint Louis, MO, USA) to reduce the undesirable peripheral effects of pilocarpine. SE was induced 30 min later by administration of pilocarpine (50 mg/kg i.p., Sigma-Aldrich). The intensity of motor seizures was classified according to Racine’s scale [23]: stage 1, immobility, eyes closed, and facial clonus; stage 2, head nodding and more severe facial clonus; stage 3, clonus of one forelimb; stage 4, rearing with bilateral forelimb clonus; and stage 5, generalized tonic–clonic seizures with rearing and falling. Within 30 min after pilocarpine injection, animals develop continuous, long-lasting generalized seizure activity (stage 4 and higher), i.e., convulsive SE. Animals that did not enter SE within 30 min were administered a second, lower dose of pilocarpine (25 mg/kg).

SE was interrupted 2 h after onset by i.p. administration of a cocktail of drugs: diazepam (10 mg/kg), phenobarbital (25 mg/kg), and scopolamine (1 mg/kg). This cocktail was administered again after 4 h. Finally, after another 4 h, rats received an i.p. administration of diazepam and scopolamine only. This procedure allows a complete stop of seizure activity [24]. To facilitate animal’s recovery and reduce the weight loss that follows SE, hydration was promoted by daily s.c. administration of 0.9% saline (1 mL) and palatable food was provided to support feeding for the next 5 days. Of the 99 rats that underwent this procedure, 15 (i.e., 15%) did not enter SE and 11 (11%) died during SE or within 24 h. The remaining 73 rats were assigned to the 3 experimental groups: vehicle, 7,8-DHF 5 mg/kg, and 7,8-DHF 10 mg/kg. Fifty have been employed for the video-EEG, behavioral and histological experiments, and the other 23 for Western blot. Allocation to groups was performed randomly on the basis of the baseline performance in the behavioral tests (see below) and on the severity of SE. Animals were killed 28 days after SE, except those used for Western blot that were killed 3, 7, or 21 days after SE. For the immunofluorescence and the Western blot studies, we also employed a group of 24 naïve animals.

### Drug Treatments

7,8-DHF (Tokyo Chemical Industry, TCI, Tokyo, Japan) was dissolved in phosphate-buffered saline (PBS 1x) containing 50% dimethylsulfoxide (DMSO). Rats received i.p. injections of 5 or 10 mg/kg 7,8-DHF or vehicle once daily for 7 consecutive days, beginning the day after SE. These regimen and doses were chosen based on previous in vivo studies demonstrating that they produce activation of central TrkB receptors, increase neurogenesis, and evoke behavioral changes in models of neurodegenerative diseases [25, 26].

### Assessment of Spontaneous Recurrent Seizures

After SE induction, animals were placed in individual cages and video monitored (24 h/day, 7 days/week) for 21 days (Videostar, Misterbianco, Catania, Italy). Frequency and severity of motor spontaneous recurrent seizures (SRSs) were recorded and scored using the scale of Racine [23], by investigators that were blind of the treatment administered to the different rats.

### Electrode Implantation

A separate subgroup of 15 animals was implanted with a bipolar electrode (PlasticsOne, Roanoke, VA, USA) in the right dorsal hippocampus 2 weeks prior to SE induction. Rats were first anesthetized using ketamine/xilazine (87 mg/kg and 15 mg/kg i.p., respectively) and anesthesia was then maintained with 2% isoflurane. Ophthalmic ointment was used for eyes lubrification. A midline incision was made in the scalp and a hole was drilled in the skull. The coordinates for electrode implantation were AP –3.9, ML –1.7 from bregma, and P –3.5 from dura [27]. A ground wire was connected to four screws secured to the skull, and the electrode was fixed with dental cement. Animals received an antibiotic prior to and after surgery (enrofloxacin, 5 mg/kg s.c.), to avoid possible infections and an analgesic drug (tramadol, 7 mg/kg s.c. daily) for 3 days after surgery.

### Video-EEG Monitoring

SE was evoked in electrode-implanted animals as described above, and rats were then randomly assigned to the different experimental groups (vehicle *n* = 5, 5 mg/kg 7,8-DHF *n* = 5, 10 mg/kg 7,8-DHF *n* = 5). The electrode was connected through a tripolar cable (PlasticsOne) to an EEG100C amplifier/MP160 Data Acquisition system (Biopac Systems, Goleta, CA, USA), paired with video cameras to record animal behavior. EEG signals were analyzed using the AcqKnowledge 5.0 software (Biopac). Seizures were detected by visual inspection of the EEG by investigators that were blind of the group to which animals belonged. An EEG seizure has been defined as a paroxysmal electrical activity characterized by (i) 3-times higher amplitude than baseline, (ii) duration of at least 5 s, and (iii) more than 5 spikes/s [28]. Video-EEG monitoring was performed 24 h/7d for 3 weeks after SE.

### Behavioral Tests

The effects of the 7,8-DHF treatment on comorbidities associated with epilepsy such as anxiety and cognition were investigated using different behavioral tests: open field (OF), elevated plus maze test (EPM), and object location task (OLT). All tests were carried out at the following time points: (i) 8–6 days before the induction of SE, at baseline; (ii) 8–10 days after SE, i.e., early phase of the disease process; and (iii) 21–23 days after SE, i.e., late phase. The early phase corresponds to the time of onset of SRSs in epileptic control animals, whereas the late phase corresponds to the chronic period, when epileptic control animals regularly experience SRSs. Tests were performed in a soundproof room, where animals were transferred 30 min before the test for acclimatization. All procedures were conducted and data analyzed by 2 investigators that were blind of the experimental conditions.

The OF test was carried out in an apparatus consisting of a square-shaped arena (82 × 82 × 40 cm). Each rat was placed in the center area and its behavior was video monitored using an infra-red video camera (DSS1000 video recording system V4.7.0041FD, AverMedia Technologies, USA) for 20 min. Recorded parameters included as follows: the total distance run by the rat, the distance run in the center quadrant of the arena (41 × 41 cm), the number of entries in the central quadrant, and the immobility time. Data were automatically measured using the ANY-Maze software (Ugo Basile, Gemonio, Varese, Italy). The OF apparatus was carefully cleaned with 70% ethanol after each test session.

The EPM test was performed as previously described [29]. The maze consisted of two open arms (50 × 10 cm) and two closed arms (50 × 10 cm) connected through a central platform (10 × 10 cm). The apparatus was 80 cm above the floor. At the start of the test, animals were placed in the central square, facing an open arm. The observation lasted 5 min. The calculated measures were the following: number of entries in open arms, number of entries in closed arms, time spent in open arms, time spent in closed arms. The EPM apparatus was carefully cleaned with 70% ethanol after each test.

The OLT was performed in the arena used for the OF test. The test consisted of three phases: habituation, training, and test. The OF test, conducted the day before OLT, was used as habituation phase. The day after habituation, the training phase was conducted by placing the rat in the arena, in which two identical objects were positioned in two adjacent corners, at 10 cm from the wall. The time of interaction of the animal with each object was recorded for 5 min. Interaction was defined as sniffing or observing the object at less than 2-cm distance [30]. After 2 h, rats were placed again in the arena, where one of the objects was moved to a different corner. Again, the time that each animal spent exploring each object was recorded for 5 min. The OLT apparatus was carefully wiped clean with 70% ethanol after each test.

### Immunofluorescence

Animals were killed 4 weeks after SE (vehicle *n* = 11, 5 mg/kg 7,8-DHF *n* = 12, 10 mg/kg 7,8-DHF *n* = 9) together with a control group of naïve animals (*n* = 7). Brains were removed and immersed in 10% neutral formalin solution (Sigma-Aldrich) for 48 h, before undergoing tissue processing (VTP 300, Bio-Optica, Milan, Italy) and paraffin-embedding. Coronal, 6-μm-thick tissue sections were cut using a Leica RM2125RT microtome across the hippocampus [27], and mounted onto polarized slides (Superfrost slides, Diapath Martinengo, Bergamo, Italy). Sections were dewaxed and rehydrated as previously described [10]: two 10-min washes in xylene (Sigma-Aldrich), 5 min in 100% ethanol, 5 min in 95% ethanol, 5 min in 80% ethanol, 5 min in distilled water.

All antigens were unmasked using a solution of citric acid and sodium citrate in a microwave oven at 750 W (5 cycles of 5 min) for NeuN; 750 W (1 cycle of 5 min) and then 350 W (2 cycles of 3 min) for glial fibrillary acidic protein (GFAP). After a wash in PBS, sections were incubated at room temperature with Triton X-100 (0.3% in 1 × PBS; Sigma-Aldrich) for 10 min, washed twice in PBS, and then incubated for 30 min with 5% bovine serum albumin and 5% serum of the species in which the secondary antibody was produced. Sections were incubated overnight at 4 °C in a humid atmosphere with a primary antibody as follows: anti-NeuN (mouse monoclonal, Immunological Science, Rome, Italy), 1:100 dilution; anti-GFAP (rabbit polyclonal, Sigma-Aldrich), 1:100. After 5 min washing in PBS, sections were incubated with Triton (as described above; 30 min), washed in PBS, and incubated with the secondary antibody, goat anti-mouse Alexa Fluor 594 (Invitrogen, Waltham, MA, USA) 1:500 for mouse primary antibodies, or goat anti-rabbit, Alexa Fluor 594 (Invitrogen) 1:500, at room temperature for 3 h. After staining, sections were washed in PBS, counterstained with 0.0001% 4,6-diamidino-2-phenylindole dihydrochloride (DAPI; Thermo Fisher Scientific, Waltham, MA, USA) for 15 min, and washed again before mounting. Coverslips were mounted using an aqueous antifading mounting gel (Sigma). The primary antibody was omitted on a subset of slices for detection of non-specific staining.

We analyzed 6 sections at 3 levels throughout the dorsal hippocampus, –2.3, –2.8, and –3.3 relative to bregma [27]. Images were captured using a 20 × objective at the level of the DG, CA3, and CA1 region using a Leica microscope (DMRA2, Leica). NeuN- and GFAP-positive pixel was measured using the Fiji (ImageJ) open-source software [31], and an algorithm tailored to measure percent of supra-threshold pixels according to the IsoData method [32]. Data were expressed as percent of positive pixels within the hilus of the DG (the region situated between the granule layer and the CA3 pyramidal neurons and remaining between the boundaries of the DG [28]), or within a rectangular frame (400 × 180 pixels) along the pyramidal layer of the CA3 and CA1 regions. Data obtained from the 6 sections examined for each rat were averaged to obtain a single estimate for each animal. The investigator who performed quantification was blinded to the experimental condition.

### Western Blot Analysis

Tissue homogenization was performed as previously described [33]. Briefly, hippocampi from naïve and 7,8 DHF-treated rats were homogenized in RIPA buffer (150 mM NaCl, 50 mM Tris–Cl (pH 8), 1% Tx-100, 0.5% Na-deoxycholate, and 0.1% SDS, protease and phosphatase inhibitors) with 25 strokes of a glass-Teflon homogenizer and centrifuged at 15,000 g, 4 °C for 15 min. The protein content was analyzed by BCA (ThermoFisher Scientific, Waltham, MA, USA). About 50 μg of proteins was separated by standard SDS-PAGE and transferred onto nitrocellulose membrane. The nitrocellulose filter was stained with Ponceau S (0.2% in 3% trichloroacetic acid) and de-stained with double distilled water for protein visualization. After 1 h of blocking with TBST (10 mM Tris/HCl, 150 mM NaCl, 0.1% Tween-20) containing 5% bovine serum albumin (Roche diagnostics, Basel, Switzerland) or skimmed powdered milk, the membranes were incubated overnight with the primary antibodies and, after extensive washing, with horseradish peroxidase-conjugated anti-rabbit or mouse secondary antibody (Bio-Rad, Hercules, CA, USA). For loading controls, membranes were stripped in acidic buffer (0.2 M glycine, 0.1% SDS, 1% Tween-20, pH 2.2) and re-probed with the appropriate antibody. In the cases where stripping was not possible, the same lysates were run simultaneously on duplicate gels, and probed with phospho- and total antibodies. Proteins were revealed by direct acquisition using the Biorad Chemidoc Imaging system by Super Signal West Chemiluminescent Substrate (ThermoFisher Scientific). Bands were quantified using ImageJ and protein levels normalized against the loading control. Phosphorylated TrkB (Y516 and Y816), AKT, ERK, and PLCγ levels were normalized against the corresponding total protein, then for loading (GAPDH). Details on the antibodies employed in Western Blot analysis are reported in Supplementary Table S1.

### Statistical Analysis and Data Availability

Statistical analyses performed in this study are reported in Supplementary Table S2. This study does not include data deposited in public repositories. Data are available on request to the corresponding author.

## Results

### Development of Spontaneous Seizures

**Fig. 1:**
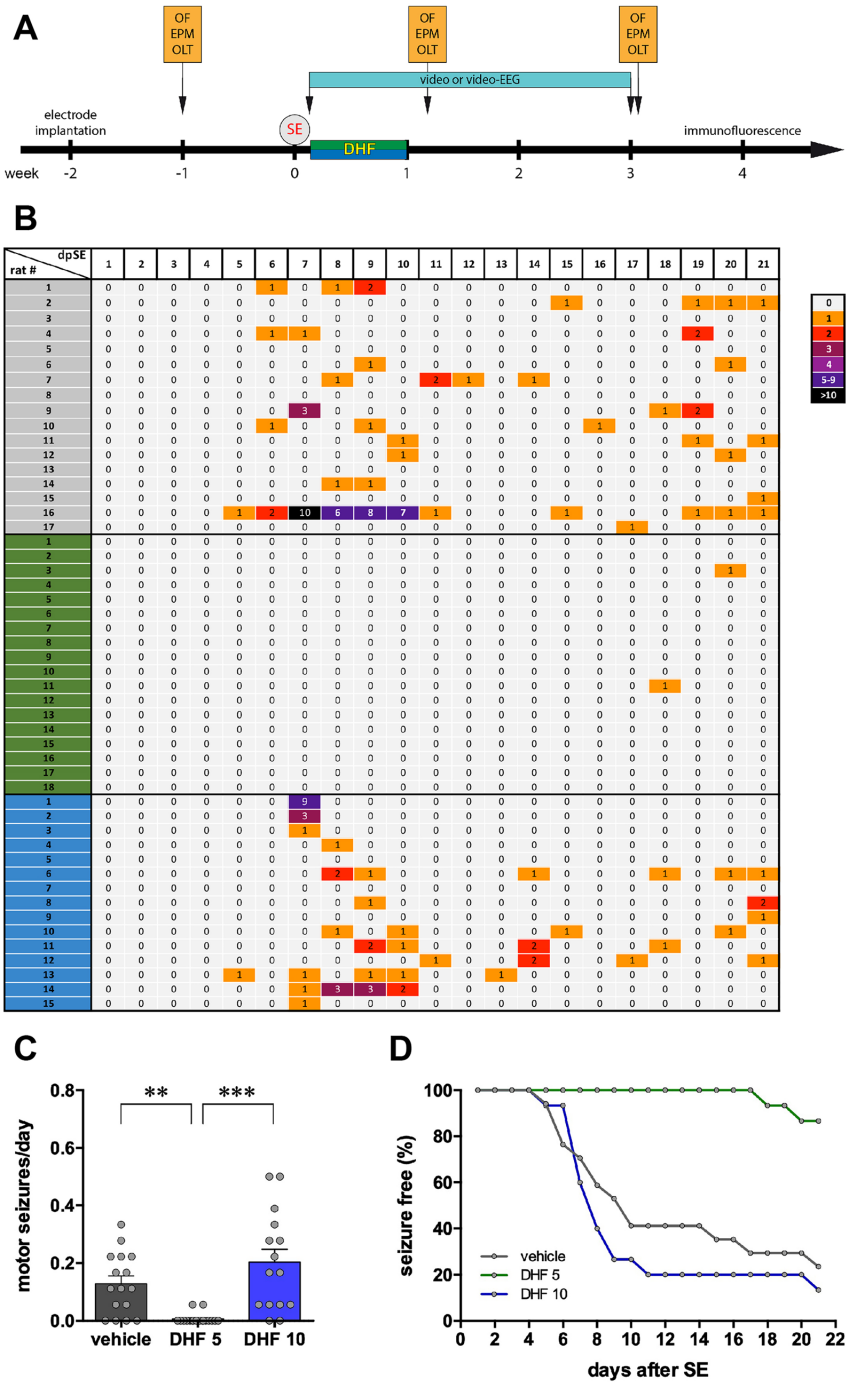
Spontaneous motor seizures. (**A**) Experimental plan. (**B**) Heat map (one rat per line) of the number of spontaneous motor seizures (class 4 or 5 according to Racine [23]) detected each day during weeks 1–3 after SE. The number of seizures per day is represented using the color code shown at the right of the panel. (**C**) Average number of spontaneous motor seizures per day in the 3 weeks after SE. Bars represent the mean ± SEM and gray dots represent data from individual animals (vehicle: *n* = 17; 7,8-DHF 5 mg/kg: *n* = 18; 7,8-DHF 10 mg/kg: *n* = 15). **p < 0.01, ***p < 0.001, Kruskal–Wallis one-way ANOVA, and post hoc Tukey’s test. (**D**) Kaplan–Meier estimates for time to first seizure. Vehicle-treated animals are represented in gray, 7,8-DHF 5 mg/kg (DHF 5) in green, and 7,8-DHF 10 mg/kg (DHF 10) in blue. Abbreviation: EPM, elevated plus maze; OF, open field; OLT, object location task; SE, status epilepticus

To test the effect of 7,8-DHF on epileptogenesis, the drug was administered daily at two different doses (5 and 10 mg/kg i.p.) for 1 week, beginning the day following SE induction (Fig. 1A). Vehicle-treated animals began experiencing EEG, non-motor seizures 6 ± 1 day after SE (*n* = 5) and motor seizures 10 ± 2 days after SE (*n* = 14, not including 3 animals that did not display any motor seizure in the 21 days of observation: Fig. 1B). Therefore, this dosing regimen covered the latency period and a very initial chronic epileptic phase with only non-generalized, non-motor seizures.

**Fig. 2:**
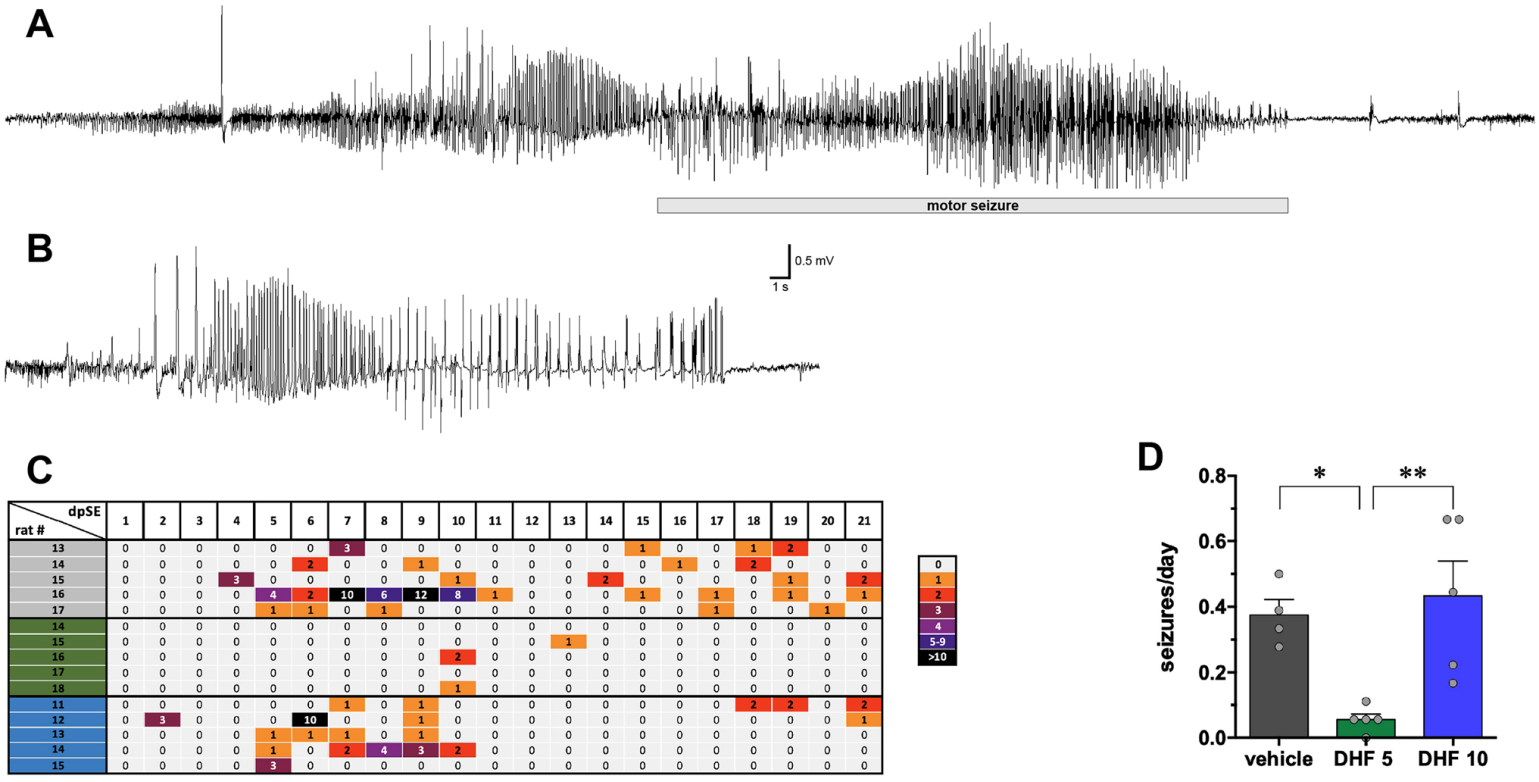
EEG. Representative EEG patterns in the hippocampus during motor (**A**) and nonmotor (**B**) seizures in vehicle-treated animals. Identical patterns were observed in animals treated with 7,8-DHF 5 or 10 mg/kg. The horizontal bar in (**A**) indicates the motor part of the seizure. (**C**) Heat map (one rat per line) of the number of spontaneous EEG seizures (motor and nonmotor) detected each day during weeks 1–3 after SE. The number of seizures per day is represented using the color code shown at the right of the panel. (**D**) Average number of spontaneous recurrent seizures (motor and nonmotor) per day in the 3 weeks after SE. Bars represent the mean ± SEM and gray dots represent data from individual animals (vehicle: *n* = 5; 7,8-DHF 5 mg/kg: *n* = 5; 7,8-DHF 10 mg/kg: *n* = 5). *p < 0.05, **p < 0.01, Kruskal–Wallis one-way ANOVA, and post hoc Tukey’s test. Vehicle-treated animals are represented in gray, 7,8-DHF 5 mg/kg (DHF 5) in green, and 7,8-DHF 10 mg/kg (DHF 10) in blue

The lower dose of 7,8-DHF almost completely prevented the occurrence of SRSs in the observation period of 21 days. Whereas, as noted above, 14 of 17 vehicle-treated rats displayed spontaneous motor seizures, only 2 of 18 rats treated with 5 mg/kg 7,8-DHF experienced each a single motor seizure, and this happened much later than in the vehicle group, i.e., 18 and 20 days after SE (Fig. 1B). Therefore, the average number of motor seizures per day was significantly lower (Fig. 1C) and the time to first seizure was highly prolonged (Fig. 1D) in 5 mg/kg 7,8-DHF-treated animals. Non-motor, EEG seizures were also dramatically attenuated by 5 mg/kg 7,8-DHF (Fig. 2). All vehicle-treated rats displayed EEG seizures. In contrast, EEG seizures were recorded in only 3 of 5 animals treated with 5 mg/kg 7,8-DHF; moreover, 2 of these 3 animals experienced a single EEG seizure, and the third just had two. Overall, the daily number of seizures was significantly reduced (Fig. 2C).

In contrast with the lower dose regimen, the higher dose did not produce any significant effect as compared with vehicle. Spontaneous motor seizures (Fig. 1B and C), time to first motor seizure (Fig. 1D), and spontaneous EEG seizures (Fig. 2C) were not significantly different in these two groups. Taken together, these data show that 7,8-DHF produces a robust and long lasting antiepileptogenic effect when administered at low but not at high doses.

### Behavioral Testing

**Fig. 3:**
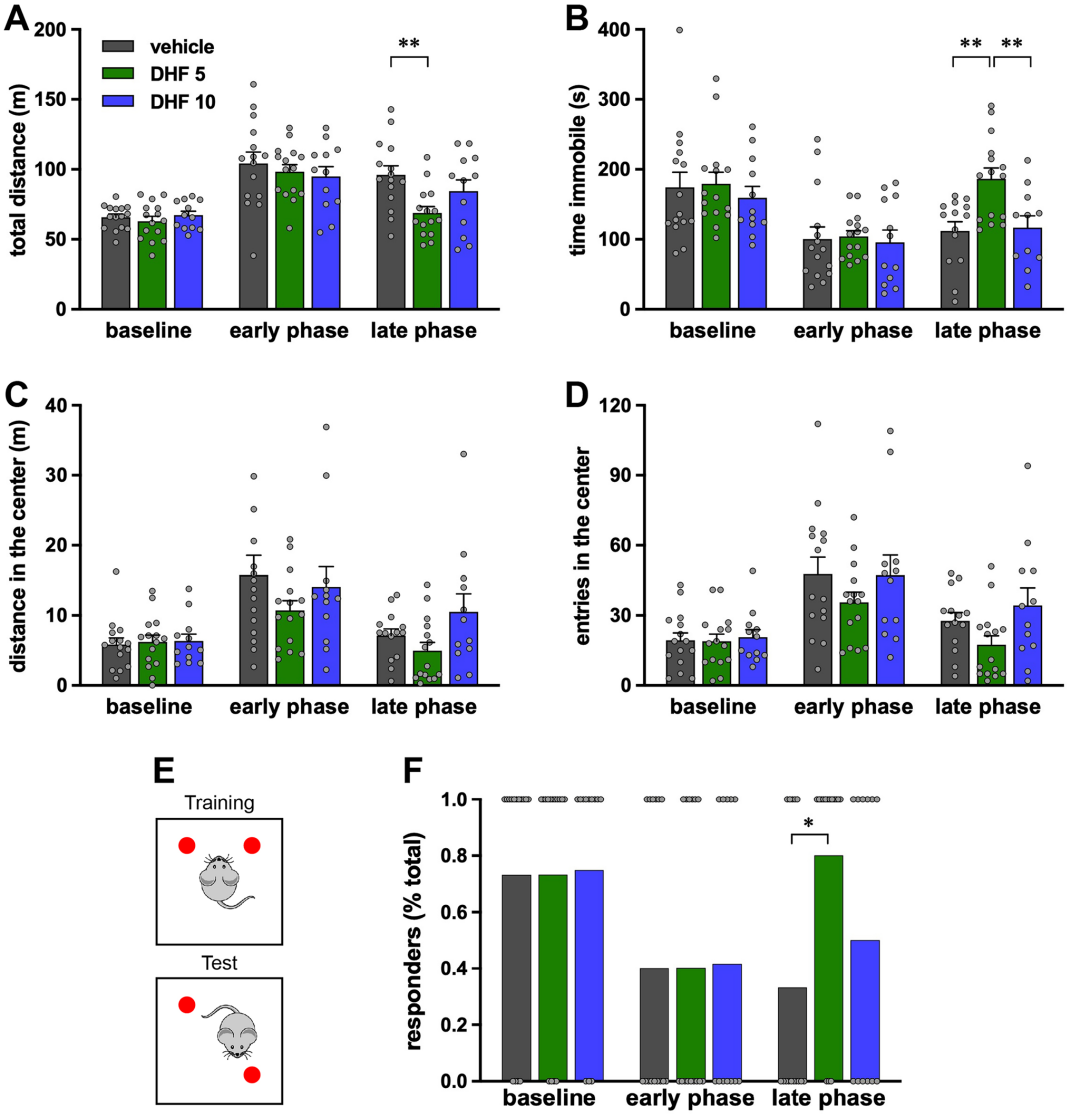
Open field (OF) test and object location task (OLT). OF was performed before SE (baseline), 8 days (early phase), or 21 days (late phase) after SE. (**A**) Total distance run by each rat. (**B**) Time spent immobile. (**C**) Distance run in center quadrants. (**D**) Number of entries in the center quadrants of the arena. Bars represent the mean ± SEM and gray dots represent data from individual animals (vehicle: *n* = 15; 7,8-DHF 5 mg/kg: *n* = 15; 7,8-DHF 10 mg/kg: *n* = 12). **p < 0.01, Kruskal–Wallis one-way ANOVA, and post hoc Tukey’s test. Vehicle-treated animals are represented in gray, 7,8-DHF 5 mg/kg (DHF 5) in green, and 7,8-DHF 10 mg/kg (DHF 10) in blue. (**E**) Schematic representation of the OLT test (see “Methods” for additional details). Twenty-four hours after the habituation phase in the empty arena, animals were allowed to explore two identical objects for 5 min (training phase). After a 2-h interval, animals were re-entered in the arena, where one object was moved in a different location, and allowed to explore the objects for another 5 min (testing phase). (**F**) Percent of animals spending more time exploring the re-positioned object. Gray dots represent individual animals. Vehicle-treated animals are in gray, 7,8-DHF 5 mg/kg (DHF 5) in green, and 7,8-DHF 10 mg/kg (DHF 10) in blue. *p < 0.01, Fisher’s exact test

We also evaluated possible effects of 7,8-DHF on epilepsy comorbidities by employing behavioral tests that explore anxiety and cognition. The OF test is used for the evaluation of both motor activity and anxiety-like behavior in rodents. Under physiological conditions, rodents spend more time in peripheral spaces than in the center of the arena. Conversely, pilocarpine-treated rats alternated periods of hyperactivity (Fig. 3A) and of freezing (Fig. 3B), spending significantly more time in the central part of the testing arena (Fig. 3C and D). This anxiety-like phenotype was partially reverted by low-dose 7,8-DHF that normalized the total distance walked by the rats and the time spent immobile in the late phase (Fig. 3A and B), but was not affected by 10 mg/kg DHF (Fig. 3A–D).

EPM is another test aimed at the evaluation of anxiety. This test is based on the rodent preference for dark and closed spaces and their fear of elevated and open spaces. Under physiological conditions, rats tend to spend more time in the closed arms of the apparatus (see baseline in Supplementary Fig. S1). Epileptic animals display a restless, anxiety-like behavior, because they spend equal or more time in the open and in the closed arms and enter the open arms much more frequently than under control, baseline conditions (Supplementary Fig. S1). 7,8-DHF did not modify this phenotype, neither at the early or at the late phase, at 5 or at 10 mg/kg (Supplementary Fig. S1).

Finally, we explored cognitive abilities using the OLT that evaluates short-term, hippocampal-dependent spatial memory [34]. At baseline, all rats displayed a clear exploratory preference for the re-located novel object, but this preference disappeared with the development of spontaneous seizures (Fig. 3E–F), indicating loss of spatial memory. However, the low (but not the high) dose of DHF reinstated the ability to distinguish the re-located object in the late phase (Fig. 3F). Taken together, these data suggest that 7,8-DHF can attenuate some epilepsy co-morbidities reinstating a more physiological behavior, when administered at low but not at high doses.

### Immunofluorescence

**Fig. 4:**
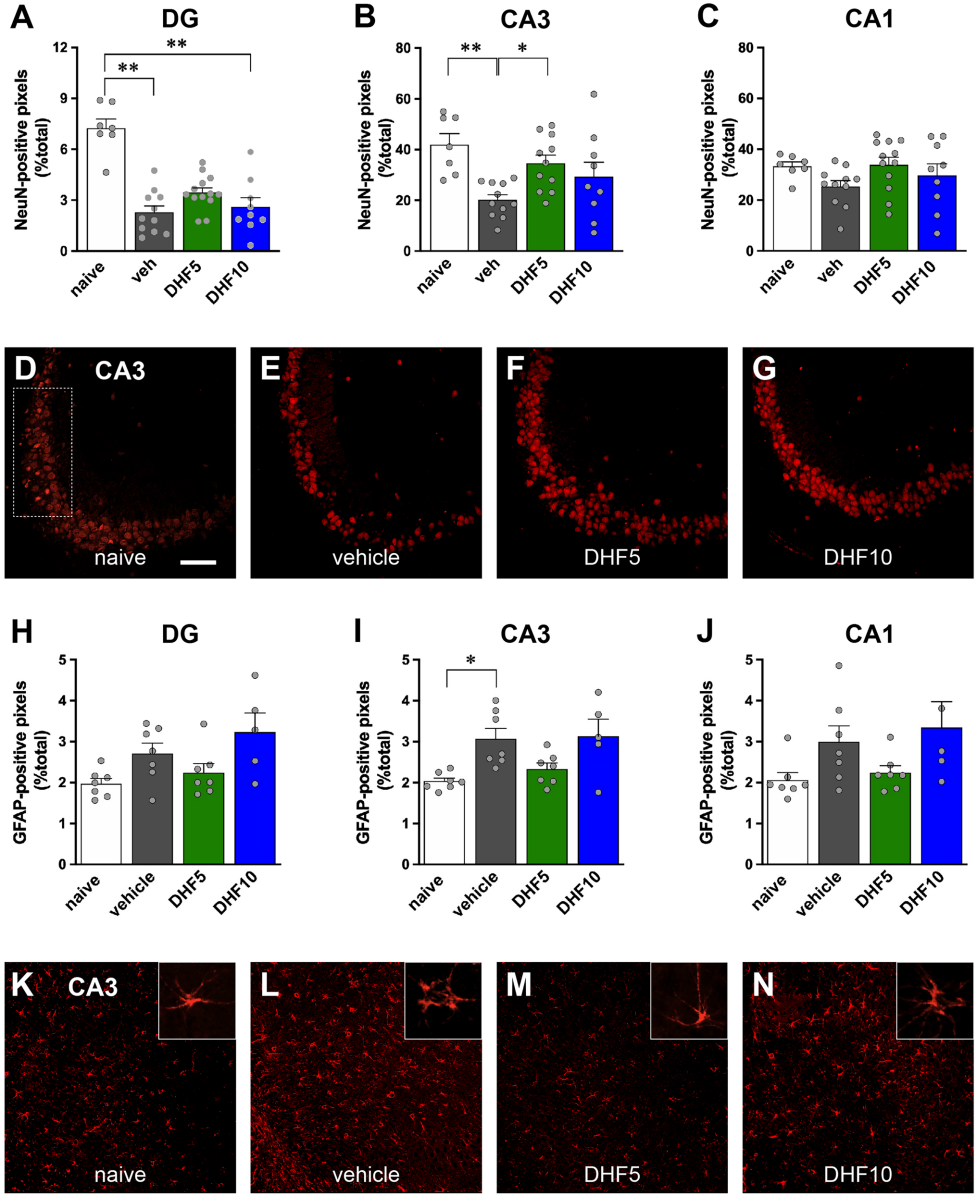
Immunohistochemical analysis. Quantification of NeuN-positive pixels in the DG (panel **A**), CA3 (**B**), and CA1 area (**C**). Data are expressed as percent of positive pixels within the hilus of the DG or within a rectangular region along the pyramidal layer of the CA3 and CA1 regions, as shown in panel (**D**). See “Methods” for details. Bars represent the mean ± SEM and gray dots represent data from individual animals (naïve: *n* = 7; vehicle: *n* = 11; 7,8-DHF 5 mg/kg: *n* = 13; 7,8-DHF 10 mg/kg: *n* = 9). Naïve animals are in white, vehicle-treated animals in gray, 7,8-DHF 5 mg/kg (DHF 5) in green, and 7,8-DHF 10 mg/kg (DHF 10) in blue. *p < 0.05, **p < 0.01, Kruskal–Wallis one-way ANOVA, and post hoc Tukey’s test. Representative sections at CA3 level of naïve (**D**), vehicle-treated (**E**), DHF 5-treated (**F**), and DHF 10-treated animals (**G**), showing neurons labeled in red with a NeuN antibody. Quantification of GFAP-positive pixels in the DG (H), CA3 (**I**), and CA1 area (**J**). Data were generated and represented like in (**A**)–(**C**). Statistical analysis was performed like in (**A**)–(**C**). Representative sections at CA3 level of naïve (**K**), vehicle-treated (**L**), DHF 5-treated (**M**), and DHF 10-treated animals (**N**), showing astrocytes labeled in red with a GFAP antibody. Horizontal bar in panel (**D**) (for all image panels) = 100 μm. Higher-magnification inserts illustrate the changes in the morphology of GFAP-positive cells

To evaluate effects on epilepsy-associated neuronal death, we performed NeuN immunofluorescence [10] in a representative subgroup of epileptic animals in comparison with naïve, non-epileptic controls. A loss in NeuN-positive cells was observed in the hilus of the dentate gyrus (DG) and in the CA3 pyramidal layer of vehicle-treated animals 1 month after pilocarpine-induced SE (Fig. 4A–E). Five mg/kg 7,8-DHF was found to partially protect from damage (Fig. 4A–G). In fact, (i) the loss of NeuN signal in the hilus of the DG was highly significant in vehicle but not in 5 mg/kg 7,8-DHF-treated pilocarpine animals (Fig. 4A); (ii) a nearly complete protection from CA3 neuronal loss was observed in animals treated with 5 mg/kg 7,8-DHF (Fig. 4B and D–G). The higher dose of 7,8-DHF did not provide a significant neuroprotection, but only a tendency to improvement in CA3 and CA1. However, no significant difference was observed between 5 and 10 mg/kg 7,8-DHF in any hippocampal subarea (Fig. 4A–G).

Epilepsy-associated astrocytosis was evaluated using GFAP immunofluorescence in sections adjected to those employed for NeuN. Consistent with previous findings [35], 1 month after pilocarpine SE, the percentage of GFAP-positive pixels in the hippocampus increased in the CA3 area and displayed a clear tendency to increase also in the hilus of the DG and in CA1 (Fig. 4H–L). In addition, many of the GFAP-positive cells in epileptic controls displayed short, thick processes, an indication of activated astrocytes (Fig. 4L insert). Once again, 5, but not 10 mg/kg 7,8-DHF prevented all these effects (Fig. 4H–N).

Taken together, these data suggest that low doses of 7,8-DHF can attenuate epilepsy-associated histological alterations.

### TrkB Receptor Phosphorylation and TrkB-Activated Intracellular Pathways

**Fig. 5:**
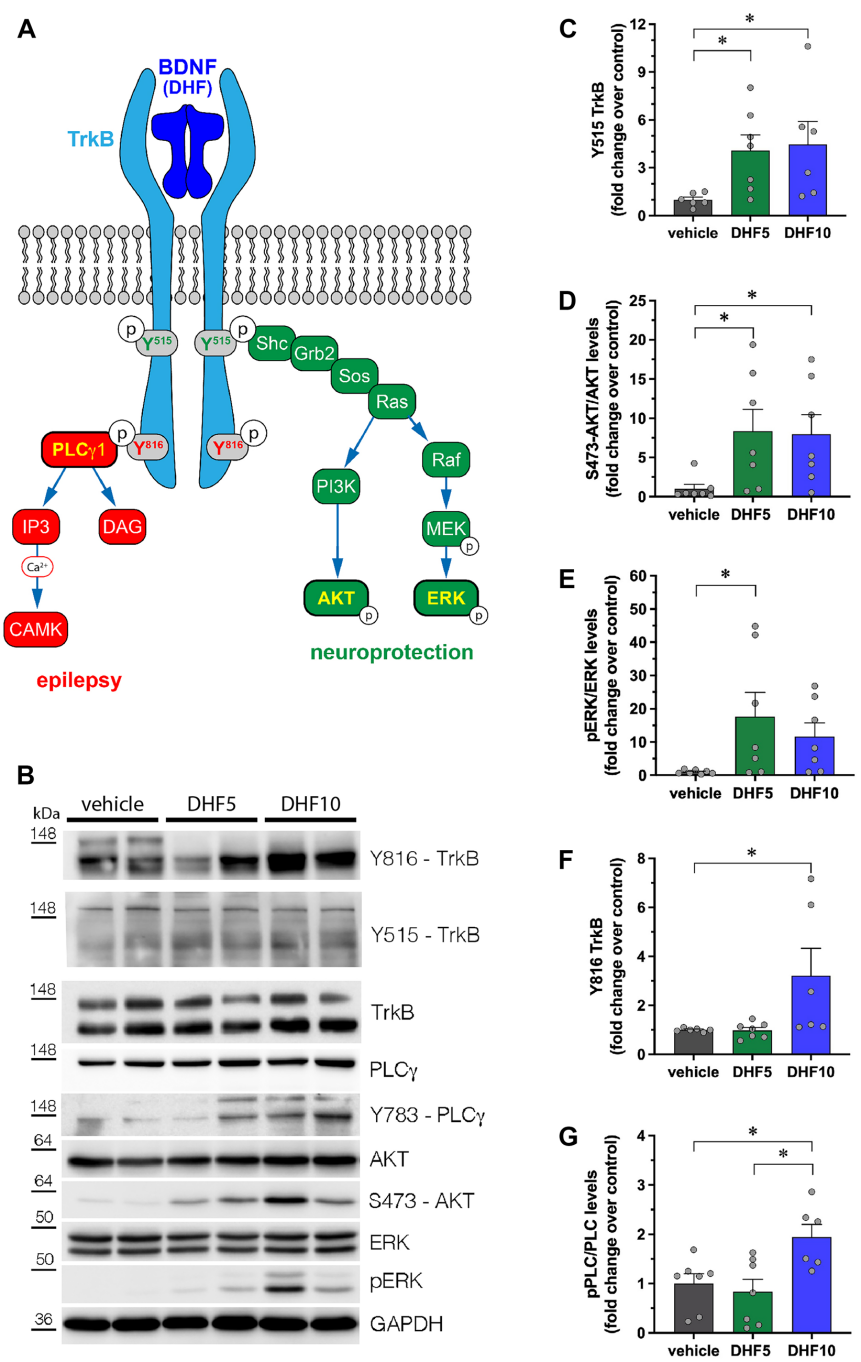
Phosphorylation of TrkB, AKT, ERK, and PLCγ proteins in the hippocampi of 7,8-DHF-treated rats. (**A**) Schematic representation of the different signaling pathways activated by BDNF (or 7,8-DHF) upon binding to TrkB receptor. (**B**) Representative western blot of the indicated proteins in extracts from hippocampi of DHF-treated rats. (**C**–**G**) Quantification of Y515 TrkB (**C**), S473-AKT (**D**), ERK (**E**), Y816 TrkB (**F**), and Y783-PLCγ (**G**) phosphorylation. Protein levels are shown as fold change over control (vehicle-treated rats). Levels of phosphorylated proteins are normalized against the corresponding total protein, then for loading (GAPDH). Vehicle: *n* = 7; 7,8-DHF 5 mg/kg: *n* = 7; 7,8-DHF 10 mg/kg: *n* = 7. *p < 0.05, Kruskal–Wallis one-way ANOVA, and post hoc Tukey’s test

All the data described above converge on the apparently paradoxical concept that a low dose of 7,8-DHF can produce beneficial effects that disappear at a higher dose. To the best of our knowledge, antioxidants have not been reported to lose their antiepileptogenic effect with an increase in dose. We therefore decided to start exploring the alternative hypothesis that may explain this observation is that these effects depend on a dose-dependent differential activation of TrkB signaling pathways. In fact, activation of TrkB by BDNF leads to receptor dimerization and auto-phosphorylation of selected tyrosines in the cytoplasmic domain. Phosphorylation of tyrosine 515 promotes association of TrkB with the Shc adaptor and activation of the PI3-kinase (PI3K)/AKT and of the Raf-MEK-ERK (i.e., MAPK/ERK) signaling pathways; phosphorylation of tyrosine 816, conversely, leads to the recruitment of phospholipase Cγ1 (PLCγ1) [36]. Whereas the former pathway has been reported to exert neuroprotective effects, the latter has been suggested to produce pro-epileptogenic effects (Fig. 5A) [37].

Therefore, we decided to test whether TrkB phosphorylation and the AKT/ERK and PLCγ1 signaling pathways were differentially activated by 7,8-DHF as a function of the dose. First, hippocampal homogenates isolated from vehicle or 7,8-DHF-treated-naïve rats were analyzed by western blot, as shown in Fig. 5B. After 7 days of treatment, we found that, whereas 7,8-DHF increased TrkB Y515, AKT, and ERK phosphorylation to similar levels at both doses (Fig. 5C–E), only the dose of 10 mg/kg increased (by ~ twofold) the levels of phosphorylated TrkB Y816 and of PLCγ1 (Fig. 5F and G). Level of all phosphorylations was not significantly different from control values after 3 days of 7,8-DHF treatment or 14 days after its discontinuation (data not shown).

**Fig. 6:**
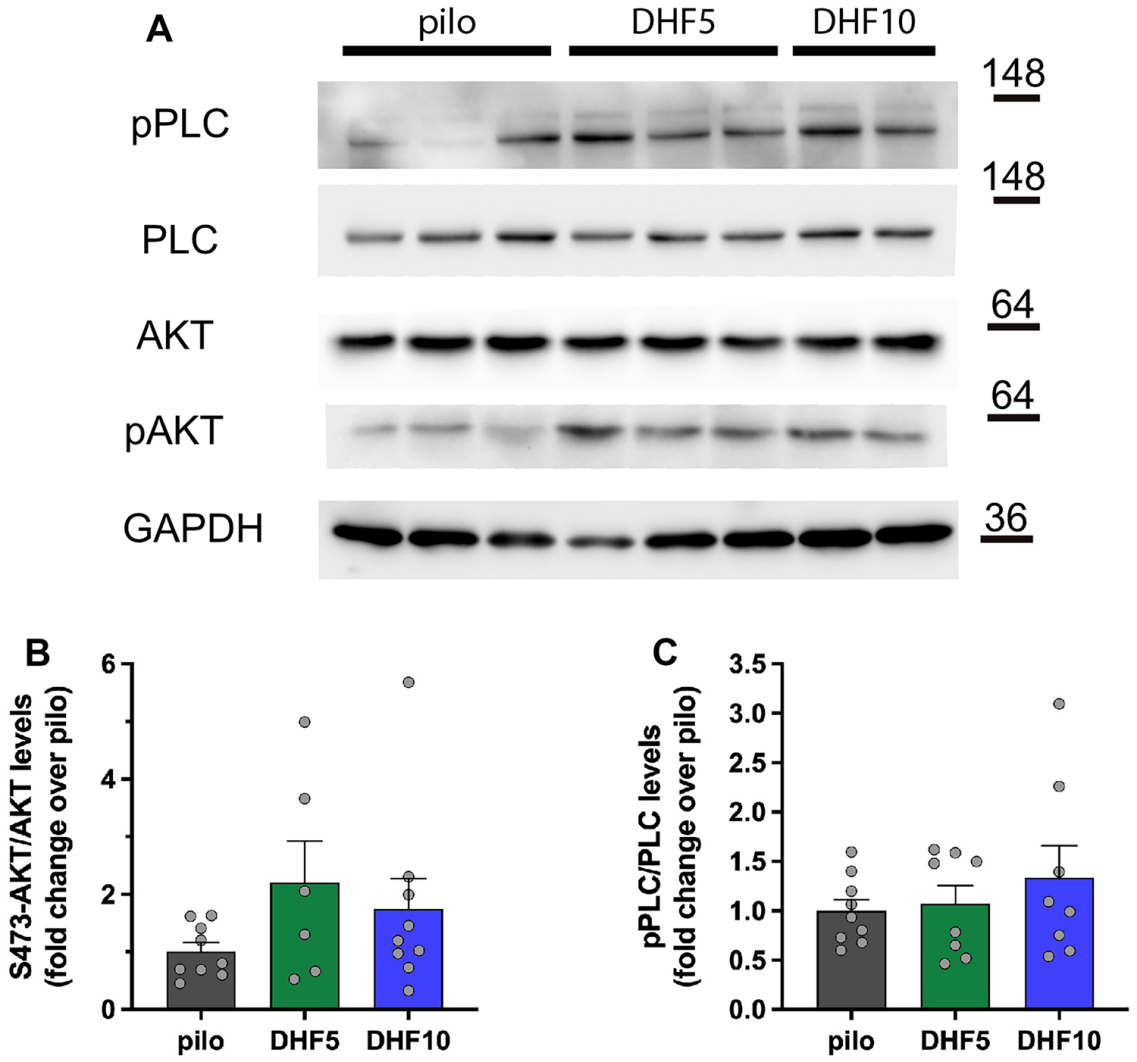
Phosphorylation of AKT and PLCγ proteins in the hippocampi of epileptic rats treated with 7,8-DHF. (**A**) Representative western blot of the indicated proteins in extracts from hippocampi of 7,8-DHF-treated rats. (**B**–**C**) Quantification of S473-AKT (**B**) and Y783-PLCγ (**C**) phosphorylation. Protein levels are shown as fold change over control (lithium-pilocarpine-treated rats). Levels of phosphorylated proteins are normalized against the corresponding total protein, then for loading (GAPDH). Pilocarpine: *n* = 9; 7,8-DHF 5 mg/kg: *n* = 9; 7,8-DHF 10 mg/kg: *n* = 9

We then attempted to extend these observations to epileptic rats. Seven days after SE, all phosphorylations were dramatically increased, and returned to near baseline levels after 21 days (Supplementary Fig. S2). We could not detect any significant further increase with 7,8-DHF treatment, except for a tendency to increased levels of phospho-AKT in both low and high dose-treated animals, and a tendency to increased levels of phosphor-PLCγ only in those treated with high-dose 7,8-DHF (Fig. 6). These changes, however, were also non-significant.

## Discussion

The main finding of this study is that low-, but not high-dose 7,8-DHF can exert strong anti-epileptogenic effects in the pilocarpine model. By using the two most commonly used 7,8-DHF treatment regimens [18], however, we found highly significant dose-dependent differences in many respects (in particular SRSs and cell death).

Flavonoids are plant metabolites that are known to produce many favorable effects in human health, due mainly, but not only, to their antioxidant and anti-inflammatory actions [38]. In fact, 7,8-DHF is a flavonoid originally known for its antioxidant properties [19], and more recently recognized as a high-affinity and selective TrkB receptor agonist. 7,8-DHF is orally bioavailable, crosses the blood–brain barrier, and has a relatively long half-life (3 h in mice, > 6 h in monkeys) [18]. Its profile of actions and its favorable pharmacokinetics have prompted a very large number of preclinical studies that highlight it as a promising treatment for many, diverse neurological and psychiatric disorders [18]. However, no study was performed thus far in epilepsy models except one [39] in which 5 mg/kg 7,8-DHF was administered for three times only (one every other day), the last time 21 days *before* kainate-induced SE. In other words, this is the first study in which 7,8-DHF was administered under clinically-relevant conditions, i.e. *after* SE and during epileptogenesis.

The puzzling observation that 7,8-DHF exerts highly favorable effects at a low, but not at a higher dose, is difficult to interpret. Antioxidant drugs are well known to exert anti-epileptogenic effects and, even if dose–response studies are not yet available, no evidence thus far supports the possibility that they may lose effect with an increase in dose [20]. However, this event cannot be excluded, because several anti-oxidant dietary phytochemicals have been reported to exert pro-oxidant activities at high doses [40]. Therefore, future studies should be designed to explore this possibility. Another hypothesis, explored in the present study, may be that the reduced antiepileptogenic effect of 7,8-DHF at relatively higher doses may be due to unwanted actions on TrkB receptors. In fact, conflicting data are available on pro- or anti-epileptogenic implications of the BDNF/TrkB system [4]. A possible explanation of this conundrum has been recently found to lay in the different TrkB receptor signaling pathways. A membrane permeable peptide comprising the HIV-1 Tat domain and a TrkB sequence, able to block PLCγ1 binding to residue 816 of TrkB, has been shown to prevent epilepsy development following intra-amygdala KA while preserving the neuroprotective effects of BDNF [17]. In contrast, intra-amygdala KA administration in mice carrying a mutation blocking the Shc-Akt signaling pathway (phenylalanine substituted for tyrosine at residue 515, TrkB^Shc/Shc^ mice) evokes similar grade SE as in WT animals, but exacerbates hippocampal neuronal death [37].

We observed beneficial effects on epileptogenesis with the low dose (5 mg/kg) of 7,8-DHF, a dose regimen at which, in naïve rats, we found a selective phosphorylation of the Y515 residue of TrkB (i.e., no phosphorylation at Y816) and a selective activation of the Shc-Akt pathway (i.e., no activation of the PLCγ1 pathway). These effects may explain the neuroprotective effect on hippocampal neurons and, together with the expected antioxidant action, contribute to the robust antiepileptogenic effect. In contrast, the higher dose of 7,8-DHF (10 mg/kg) induces TrkB phosphorylation at both Y515 and Y816, recruits the PLCγ1 pathway, and, by doing so, it may oppose the neuroprotective and anti-epileptogenic antioxidant effects. It is unclear how 7,8-DHF may differentially activate different TrkB signaling pathways in a dose-dependent manner. Because it is known to bind the extracellular domain of the receptor at a different site in comparison with BDNF [16], it may be hypothesized that the conformational changes induced by low 7,8-DHF doses in the intracellular domain prompt a preferential phosphorylation of tyrosine 515.

However, we were unable to confirm these findings in lithium-pilocarpine rats. It should be kept in mind that this was a very difficult experiment, because of multiple confounding factors. First, lithium-pilocarpine SE per se increases TrkB activation, making changes induced by 7,8-DHF proportionally smaller. Second, recent seizures can further boost TrkB receptor activation for a relatively long time. Therefore, levels of phosphorylations are expected to undergo remarkable oscillations in time in epileptic animals. Seven days after SE, some of the animals treated with vehicle- or 10 mg/kg 7,8-DHF (but not those treated with 5 mg/kg — Fig. 2) may have already experienced EEG seizures. All these factors notwithstanding, our hypothesis remains undemonstrated at this time, and further studies will be required to clarify the reason of the paradoxically greater effects of low-dose 7,8-DHF.

It seems instead unlikely that the effects of 7,8-DHF depend on TrkB receptor internalization. First, as compared with BDNF, 7,8-DHF has been shown to induce a much slower TrkB internalization and a much longer-lasting phosphorylation, not inducing its ubiquitination or degradation [41]. Incidentally, these findings suggest that 7,8-DHF and BDNF activate TrkB with different mechanisms, indirectly supporting the above hypothesis that the patterns of activation of intracellular pathways may also differ. Second, internalization would lead to an antagonist-like effect, switching off all TrkB-activated signaling pathways and all TrkB-dependent effects, a condition under which one would expect an anti-epileptogenic effect [16]. Internalization would be stronger with the high dose of 7,8-DHF, which would therefore produce a more robust anti-epileptogenic effect than the low dose. However, we observed the opposite. Third, a reduced activation of signaling pathways would be expected in case of internalization. Not only this was not the case, but the higher dose proved even more effective than the lower dose in activating signaling pathways.

The primary outcome measure in this study was the frequency and severity of spontaneous seizures. However, we also investigated the impact of the treatment on co-morbidities, in particular anxiety and cognition. Based on OF (but not EPM) and on OLT, we observed an attenuation of these co-morbidities in animals treated with 7,8-DHF at low, but again not at high doses. However, these effects were partial for anxiety, because only a few parameters of the OF were corrected, and all appeared only in the late, chronic phase of the disease, while anxiety traits and cognitive impairments were observed in all animals (including those treated with low-dose 7,8-DHF) in the early phase, i.e., at the time when vehicle-treated animals begin experiencing SRSs. Several behavioral alterations have been observed to follow epileptogenic insults (SE or traumatic brain injury, TBI) in animal models, but the majority of these alterations cannot predict which animals will subsequently become epileptic (i.e., will display SRSs) and which will not [42–45]. In the chronic course of epilepsy, these behavioral alterations are generally maintained [43–45], and this was the case also in the present study. Therefore, the observation that animals treated with low-dose 7,8-DHF had improvements at late time points may be attributed to the fact that they did not (or very marginally did) experience SRSs. In other words, SRSs seem to sustain anxiety-like behavior and cognitive impairment in our experimental settings, because these behavioral alterations tend to attenuate in time in animals receiving a treatment that prevents seizure occurrence.

In conclusion, considering its pharmacological properties and context of use (good pharmacokinetics and tolerability, antioxidant effects, profile of actions on the TrkB receptor, and prospective short-term administration following an epileptogenic insult), 7,8-DHF may represent a candidate for a preventive, anti-epileptogenic therapy or, probably more realistically, a template for developing an effective and well-tolerated anti-epileptogenic drug. In the prospect of clinical translation of 7,8-DHF, the present data suggest that an accurate dose titration would be needed, and this may imply identification of an optimal window of plasma concentrations and a better understanding of the mechanism of 7,8-DHF bell-shaped anti-epileptogenic effect.

## Supplementary Information

Below is the link to the electronic supplementary material.

Supplementary file1 (PDF 455 KB)

Supplementary file2 (PDF 458 KB)

Supplementary file3 (PDF 557 KB)

Supplementary file4 (PDF 456 KB)

Supplementary file5 (PDF 457 KB)

Supplementary file6 (PDF 458 KB)

Supplementary file7 (PDF 459 KB)

Supplementary file8 (PDF 460 KB)

Supplementary file9 (PDF 460 KB)

Supplementary file10 (PDF 461 KB)

Supplementary file11 (PDF 462 KB)

Supplementary file12 (PDF 463 KB)
