# Supplementary material for: Low-dose 7,8-Dihydroxyflavone Administration After Status Epilepticus Prevents Epilepsy Development
Source: Neurotherapeutics. 2022 Sep 30;19(6):1951–65. doi: 10.1007/s13311-022-01299-4 (PMC9723075; doi:10.1007/s13311-022-01299-4)
Supplement: Supplementary file 3 — Supplementary file3 (PDF 557 KB) [file 13311_2022_1299_MOESM3_ESM.pdf]

Low-dose 7,8-Dihydroxyflavone Administration after Status Epilepticus Prevents Epilepsy Development

Annunziata Guarino,[1*] Barbara Bettegazzi,[2*] Nimra Aziz,[1] Mario Barbieri,[1] Daniela Bochicchio,[1] Lucia Crippa,[2] Pietro Marino,[1] Maddalena Sguizzato,[1] Marie Soukupova,[1] Silvia Zucchini,[1,3] Michele Simonato[1,4]

[1] Department of Neuroscience and Rehabilitation, University of Ferrara, via Fossato di Mortara 70, 44121 Ferrara, Italy
[2] University Vita-Salute San Raffaele, via Olgettina 58, 20132 Milan, Italy
[3] Laboratory of Technologies for Advanced Therapy (LTTA), Technopole of Ferrara, Ferrara, Italy
[4] Division of Neuroscience, IRCCS San Raffaele Hospital, via Olgettina 60, 20132 Milan, Italy

[*] These authors contributed equally to this work.

Address correspondence to
Silvia Zucchini
Department of Neuroscience and Rehabilitation
University of Ferrara
via Fossato di Mortara 70
44121 Ferrara
Italy
Email: silvia.zucchini@unife.it

Supplementary Table 1. Antibodies employed in Western Blot.

Antibody	Product no.	Concentration	Blocking	Producer
S473-AKT	4060	1:1000	5% BSA in TBST	Cell Signaling Technology
AKT	4691	1:5000	5% BSA in TBST	Cell Signaling Technology
Phospho-ERK	4370	1:1000	5% BSA in TBST	Cell Signaling Technology
ERK	9102	1:1000	5% BSA in TBST	Cell Signaling Technology
alpha Tubulin	T9026	1:6000	5% Milk in TBST	Merck
GAPDH (14C10)	2118	1:1000	5% Milk in TBST	Cell Signaling Technology
PLCγ	5690	1:1000	5% BSA in TBST	Cell Signaling Technology
Phospho-PLCγ Tyr783	2821	1:1000	5% BSA in TBST	Cell Signaling Technology
TrkB	4603	1:1000	5% BSA in TBST	Cell Signaling Technology
TrkB Y816	ABN1381	1:1000	5% Milk in TBST	Merck
TrkB Y515	LS-C336054-50	1:1000	5% Milk in TBST	LSBio

Supplementary Table 2. Statistical information.

Figures 1-4

	Experiment	Outcome measures	Time point	Test	Treatment	Mean Diff	95,00% CI of diff	Below threshold	Summary	Adjusted P Value
Seizures	Mean beavioural Seizure/day		1-21 dpSE	Tukey's multiple comparisons test	vehicle vs. DHF 5	0.1223	0,02821 to 0,2164	Yes	**	0.008
					vehicle vs. DHF 10	-0.07523	-0,1736 to 0,02319	No	ns	0.1646
					DHF 5 vs. DHF 10	-0.1975	-0,2933 to -0,1018	Yes	****	<0,0001
	Mean EEG Seizure/day		1-21 dpSE	Tukey's multiple comparisons test	vehicle vs. DHF 5	0.3194	0,04204 to 0,5968	Yes	*	0.0248
					vehicle vs. DHF 10	-0.05833	-0,3357 to 0,2191	No	ns	0.8395
					DHF 5 vs. DHF 10	-0.3778	-0,6393 to -0,1162	Yes	**	0.0064
Behavioral Tests	Open field	total distance travelled	baseline	Tukey's multiple comparisons test	veh vs. DHF 5	2.853	-7,417 to 13,12	No	ns	0.7695
					veh vs. DHF 10	-1.362	-10,58 to 7,860	No	ns	0.9272
					DHF 5 vs. DHF 10	-4.215	-15,42 to 6,989	No	ns	0.6224
			early phase	Tukey's multiple comparisons test	veh vs. DHF 5	5.891	-18,00 to 29,78	No	ns	0.8124
					veh vs. DHF 10	9.517	-17,32 to 36,36	No	ns	0.6556
					DHF 5 vs. DHF 10	3.626	-18,22 to 25,48	No	ns	0.9083
			late phase	Tukey's multiple comparisons test	veh vs. DHF 5	27.25	7,738 to 46,77	Yes	**	0.0051
					veh vs. DHF 10	11.82	-14,03 to 37,67	No	ns	0.4958
					DHF 5 vs. DHF 10	-15.43	-39,15 to 8,286	No	ns	0.2466
		Time immobile	baseline	Tukey's multiple comparisons test	veh vs. DHF 5	-4.873	-72,35 to 62,60	No	ns	0.9824
					veh vs. DHF 10	15.13	-51,83 to 82,08	No	ns	0.8405
					DHF 5 vs. DHF 10	20	-37,57 to 77,56	No	ns	0.6664
			early phase	Tukey's multiple comparisons test	veh vs. DHF 5	-3.733	-51,64 to 44,18	No	ns	0.9789
					veh vs. DHF 10	4.8	-56,24 to 65,84	No	ns	0.979
					DHF 5 vs. DHF 10	8.533	-41,51 to 58,58	No	ns	0.8992
			late phase	Tukey's multiple comparisons test	veh vs. DHF 5	-64.13	-119,3 to -8,921	Yes	*	0.0203
					veh vs. DHF 10	-22.31	-114,6 to 69,97	No	ns	0.8101
					DHF 5 vs. DHF 10	41.82	-49,99 to 133,6	No	ns	0.4842
		distance in the center	baseline	Tukey's multiple comparisons test	veh vs. DHF 5	-0.3787	-3,751 to 2,994	No	ns	0.9584
					veh vs. DHF 10	-0.496	-3,897 to 2,905	No	ns	0.9298
					DHF 5 vs. DHF 10	-0.1174	-3,584 to 3,349	No	ns	0.9961
			early phase	Tukey's multiple comparisons test	veh vs. DHF 5	5.043	-2,924 to 13,01	No	ns	0.2687
					veh vs. DHF 10	1.676	-8,363 to 11,72	No	ns	0.9092
					DHF 5 vs. DHF 10	-3.366	-11,57 to 4,842	No	ns	0.5528
			late phase	Tukey's multiple comparisons test	veh vs. DHF 5	5.21	-3,383 to 13,80	No	ns	0.2931
					veh vs. DHF 10	-0.34	-10,49 to 9,813	No	ns	0.9962
					DHF 5 vs. DHF 10	-5.55	-12,90 to 1,804	No	ns	0.1573
		entries in the center	baseline	Tukey's multiple comparisons test	veh vs. DHF 5	0.4	-10,51 to 11,31	No	ns	0.9955
					veh vs. DHF 10	-1.333	-12,68 to 10,01	No	ns	0.9538
					DHF 5 vs. DHF 10	-1.733	-12,90 to 9,433	No	ns	0.9208
			early phase	Tukey's multiple comparisons test	veh vs. DHF 5	12.13	-8,894 to 33,16	No	ns	0.3355
					veh vs. DHF 10	0.5667	-27,71 to 28,84	No	ns	0.9986
					DHF 5 vs. DHF 10	-11.57	-36,67 to 13,54	No	ns	0.4778
			late phase	Tukey's multiple comparisons test	veh vs. DHF 5	17.8	-5,071 to 40,67	No	ns	0.1459
					veh vs. DHF 10	0.8667	-26,75 to 28,49	No	ns	0.9966
					DHF 5 vs. DHF 10	-16.93	-38,59 to 4,726	No	ns	0.1408
	Elevated plus maze	Time open arms	baseline	Tukey's multiple comparisons test	veh vs. DHF 5	1.867	-51,89 to 55,62	No	ns	0.9963
					veh vs. DHF 10	-0.3333	-57,24 to 56,57	No	ns	0.9999
					DHF 5 vs. DHF 10	-2.2	-58,22 to 53,82	No	ns	0.9952
			early phase	Tukey's multiple comparisons test	veh vs. DHF 5	-3.505	-57,26 to 50,25	No	ns	0.9869
					veh vs. DHF 10	-22.07	-78,98 to 34,83	No	ns	0.6281
					DHF 5 vs. DHF 10	-18.57	-74,59 to 37,46	No	ns	0.7117
			late phase	Tukey's multiple	veh vs. DHF 5	1.419	-52,33 to 55,17	No	ns	0.9978

				comparisons test	veh vs. DHF 10	-6.798	-63,70 to 50,11	No	ns	0.9566
					DHF 5 vs. DHF 10	-8.217	-64,24 to 47,81	No	ns	0.9354
		Time closed arms	baseline	Tukey's multiple comparisons test	veh vs. DHF 5	-2.719	-64,29 to 58,85	No	ns	0.994
					veh vs. DHF 10	6.131	-59,05 to 71,31	No	ns	0.9729
					DHF 5 vs. DHF 10	8.85	-55,32 to 73,02	No	ns	0.9426
			early phase	Tukey's multiple comparisons test	veh vs. DHF 5	29.9	-31,67 to 91,47	No	ns	0.4835
					veh vs. DHF 10	16.33	-48,84 to 81,51	No	ns	0.823
					DHF 5 vs. DHF 10	-13.57	-77,73 to 50,60	No	ns	0.8705
			late phase	Tukey's multiple comparisons test	veh vs. DHF 5	-15.57	-77,13 to 46,00	No	ns	0.8202
					veh vs. DHF 10	-35.58	-100,8 to 29,59	No	ns	0.4001
					DHF 5 vs. DHF 10	-20.02	-84,18 to 44,15	No	ns	0.7398
		Entries open arms	baseline	Tukey's multiple comparisons test	veh vs. DHF 5	0.2619	-3,968 to 4,492	No	ns	
					veh vs. DHF 10	0.4286	-4,049 to 4,906	No	ns	0.9719
					DHF 5 vs. DHF 10	0.1667	-4,242 to 4,575	No	ns	0.9956
			early phase	Tukey's multiple comparisons test	veh vs. DHF 5	-0.1667	-4,397 to 4,063	No	ns	0.9952
					veh vs. DHF 10	-2.167	-6,645 to 2,311	No	ns	0.4861
					DHF 5 vs. DHF 10	-2	-6,408 to 2,408	No	ns	0.53
			late phase	Tukey's multiple comparisons test	veh vs. DHF 5	-0.1619	-4,392 to 4,068	No	ns	0.9955
					veh vs. DHF 10	0.5714	-3,906 to 5,049	No	ns	0.9507
					DHF 5 vs. DHF 10	0.7333	-3,675 to 5,142	No	ns	0.9176
		Entries closed arms	baseline	Tukey's multiple comparisons test	veh vs. DHF 5	-2.057	-6,662 to 2,548	No	ns	0.5172
					veh vs. DHF 10	0.2262	-4,047 to 4,500	No	ns	0.9904
					DHF 5 vs. DHF 10	2.283	-1,717 to 6,283	No	ns	0.345
			early phase	Tukey's multiple comparisons test	veh vs. DHF 5	-2.233	-5,538 to 1,071	No	ns	0.2319
					veh vs. DHF 10	-3.667	-7,344 to 0,01116	No	ns	0.0508
					DHF 5 vs. DHF 10	-1.433	-4,865 to 1,998	No	ns	0.5554
			late phase	Tukey's multiple comparisons test	veh vs. DHF 5	-0.2571	-5,928 to 5,413	No	ns	0.9931
					veh vs. DHF 10	2.643	-3,625 to 8,910	No	ns	0.5501
					DHF 5 vs. DHF 10	2.9	-3,255 to 9,055	No	ns	0.4767
	Object location task			Fisher's exact test				Yes	*	0.0253
Histology	GFAP		CA1	Dunn's multiple comparisons test	veh vs. Naive	8.714		No	ns	0.1981
					DHF 5 vs. Naive	3.643		No	ns	>0,9999
					DHF 10 vs. Naive	10.93		No	ns	0.088
					DHF 5 vs. veh	-5.071		No	ns	>0,9999
					DHF 10 vs. veh	2.214		No	ns	>0,9999
					DHF 10 vs. DHF 5	7.286		No	ns	0.6223
			CA3	Dunn's multiple comparisons test	veh vs. Naive	12.29		Yes	*	0.0159
					DHF 5 vs. Naive	5.214		No	ns	>0,9999
					DHF 10 vs. Naive	11.9		Yes	*	0.0472
					DHF 5 vs. veh	-7.071		No	ns	0.5018
					DHF 10 vs. veh	-0.3857		No	ns	>0,9999
					DHF 10 vs. DHF 5	6.686		No	ns	0.8125
			DG	Dunn's multiple comparisons test	veh vs. Naive	8.143		No	ns	0.2782
					DHF 5 vs. Naive	3.214		No	ns	>0,9999
					DHF 10 vs. Naive	10.84		No	ns	0.0927
					DHF 5 vs. veh	-4.929		No	ns	>0,9999
					DHF 10 vs. veh	2.7		No	ns	>0,9999
					DHF 10 vs. DHF 5	7.629		No	ns	0.5307
	NeuN		CA1	Dunn's multiple comparisons test	veh vs. Naive	-9.221		No	ns	0.5664
					DHF 5 vs. Naive	1.643		No	ns	>0,9999
					DHF 10 vs. Naive	-3.302		No	ns	>0,9999
					DHF 5 vs. veh	10.86		No	ns	0.1347
					DHF 10 vs. veh	5.919		No	ns	>0,9999

					DHF 10 vs. DHF 5	-4.944		No	ns	>0,9999
			CA3	Dunn's multiple comparisons test	veh vs. Naive	-19.4		Yes	**	0.0026
					DHF 5 vs. Naive	-5.524		No	ns	>0,9999
					DHF 10 vs. Naive	-11.63		No	ns	0.2573
					DHF 5 vs. veh	13.88		Yes	*	0.0213
					DHF 10 vs. veh	7.768		No	ns	0.7775
					DHF 10 vs. DHF 5	-6.111		No	ns	>0,9999
			DG	Dunn's multiple comparisons test	veh vs. Naive	-23.43		Yes	***	0.0002
					DHF 5 vs. Naive	-14.35		No	ns	0.0529
					DHF 10 vs. Naive	-21.43		Yes	**	0.0017
					DHF 5 vs. veh	9.077		No	ns	0.3483
					DHF 10 vs. veh	2		No	ns	>0,9999
					DHF 10 vs. DHF 5	-7.077		No	ns	0.9761

Figure 5

		Mean	S. E. M	C. I	P	Adjusted P value
pPLC	naive	1	0.1994	0.5120, 1.488	0.0177	naive vs. 5mg/Kg >0.9999
	DHF 5mg/Kg	0.8350	0.2511	0.2206, 1.449		naive vs. 10mg/Kg 0.0429
	DHF 10 mg/kg	1.941	0.2594	1.275, 2.608		
pERK	naive	1	0.2299	0.4374, 1.563	0.0157	naive vs. 5mg/Kg 0.0250
	DHF 5mg/Kg	17.72	7.172	0.1672, 35.27		naive vs. 10mg/Kg 0.0449
	DHF 10 mg/kg	11.72	4.032	1.857, 21.59		
pAKT	naive	1	0.5507	-0.3476, 2.348	0.0068	naive vs. 5mg/Kg 0.0172
	DHF 5mg/Kg	8.350	2.795	1.512, 15.19		naive vs. 10mg/Kg 0.0221
	DHF 10 mg/kg	7.962	2.481	1.890, 14.03		
Y515 TrkB	naive	1	0.1691	0.5653, 1.435	0.0091	naive vs. 5mg/Kg 0.0227
	DHF 5mg/Kg	4.084	0.9722	1.705, 6.462		naive vs. 10mg/Kg 0.0239
	DHF 10 mg/kg	4.469	1.442	0.7615, 8.176		
Y816 TrkB	naive	1	0.02981	0.9234, 1.077	0.0113	naive vs. 5mg/Kg >0.9999
	DHF 5mg/Kg	0.9797	0.1202	0.6856, 1.274		naive vs. 10mg/Kg 0.0389
	DHF 10 mg/kg	3.217	1.114	0.3542, 6.079		

Figure 6

		Mean	S. E. M	C. I	P	Adjusted P value
pPLC	naive	1	0.1142	0.7366, 1.263	0.8931	naive vs. 5mg/Kg >0.9999
	DHF 5mg/Kg	1.348	0.3170	0.6174, 2.079		naive vs. 10mg/Kg >0.9999
	DHF 10 mg/kg	1.339	0.3179	0.5878, 2.091		

pAKT	naive	1	0.1527	0.6584, 1.362	0.2724	naive vs. 5mg/Kg 0.2247
	DHF 5mg/Kg	3.169	1.147	0.3618, 5.975		naive vs. 10mg/Kg 0.6416
	DHF 10 mg/kg	1.743	0.5325	0.5153, 2.971		

Supplementary Figure S2

		Mean	S. E. M	C. I	P	Adjusted P value
pPLC	naive	1	0.3379	0.06194, 1.938	0.0396	naive vs. 5mg/Kg 0.0297
	Pilo 7 days	3.967	0.9354	1.370, 6.564		naive vs. 10mg/Kg 0.5830
	Pilo 21 days	2.280	0.6387	-0.4683, 5.028		
pERK	naive	1	0.2789	0.2257, 1.774	0.0511	naive vs. 5mg/Kg 0.0452
	Pilo 7 days	2.316	0.5071	0.9079, 3.724		naive vs. 10mg/Kg 0.5776
	Pilo 21 days	1.662	0.2547	-1.573, 4.898		
pAKT	naive	1	0.4148	-0.1517, 2.152	0.0033	naive vs. 5mg/Kg 0.0237
	Pilo 7 days	4.317	0.5164	2.883, 5.751		naive vs. 10mg/Kg 0.0277
	Pilo 21 days	4.765	0.8275	1.205, 8.326		
Y515 TrkB	naive	1	0.08593	0.7614, 1.239	0.0010	naive vs. 5mg/Kg 0.0460
	Pilo 7 days	2.512	0.3813	1.454, 3.571		naive vs. 10mg/Kg 0.7975
	Pilo 21 days	0.8244	0.1075	0.3621, 1.287		
Y816 TrkB	naive	1	0.09904	0.7250, 1.275	0.0149	naive vs. 5mg/Kg 0.0237
	Pilo 7 days	2.407	0.4324	1.207, 3.608		naive vs. 10mg/Kg 0.1281
	Pilo 21 days	2.042	0.2190	1.100, 2.984		

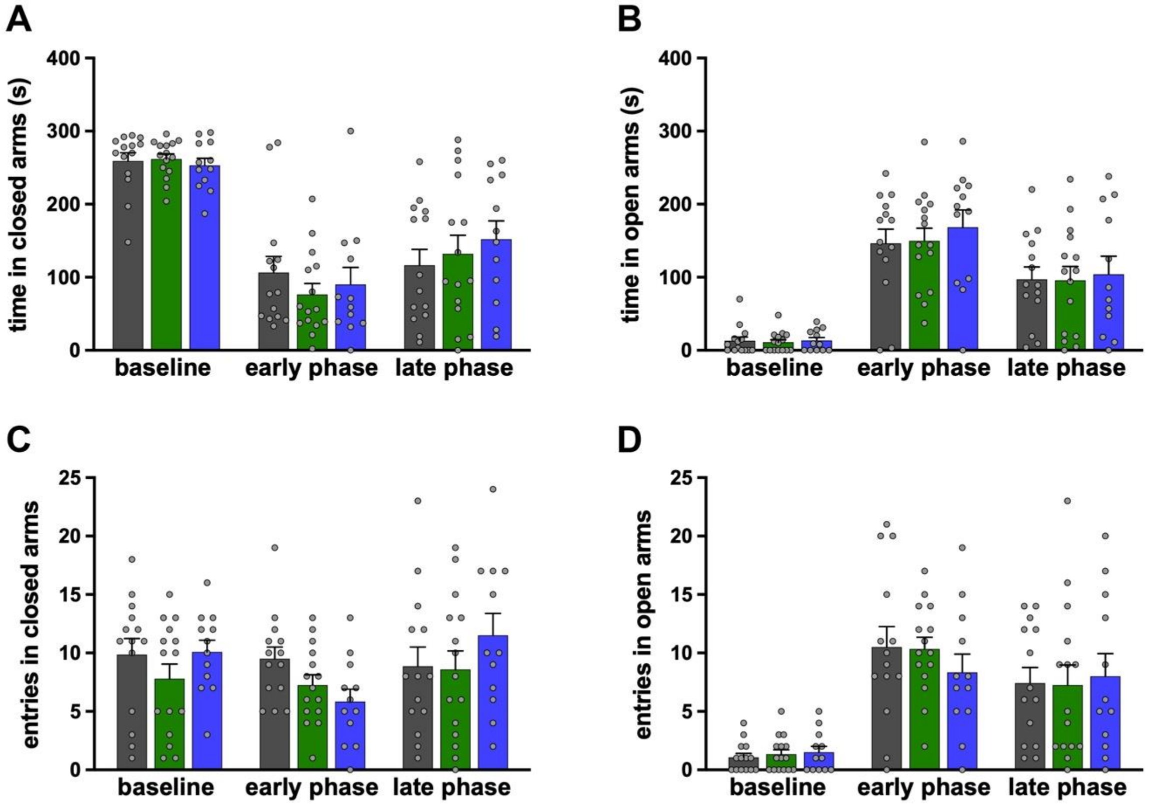

Supplementary Figure 1. Elevated plus maze (EPM) test, performed before SE (baseline), 8 days (early phase) or 21 days (late phase) after SE. (**A**) Time spent in closed arms. (**B**) Time spent in open arms. (**C**) Number of entries in closed arms. (**D**) Number of entries in open arms. Bars represent the mean ± SEM and gray dots represent data from individual animals. Vehicle-treated animals are represented in gray, 7,8-DHF 5 mg/kg (DHF 5) in green, 7,8-DHF 10 mg/kg (DHF 10) in blue.

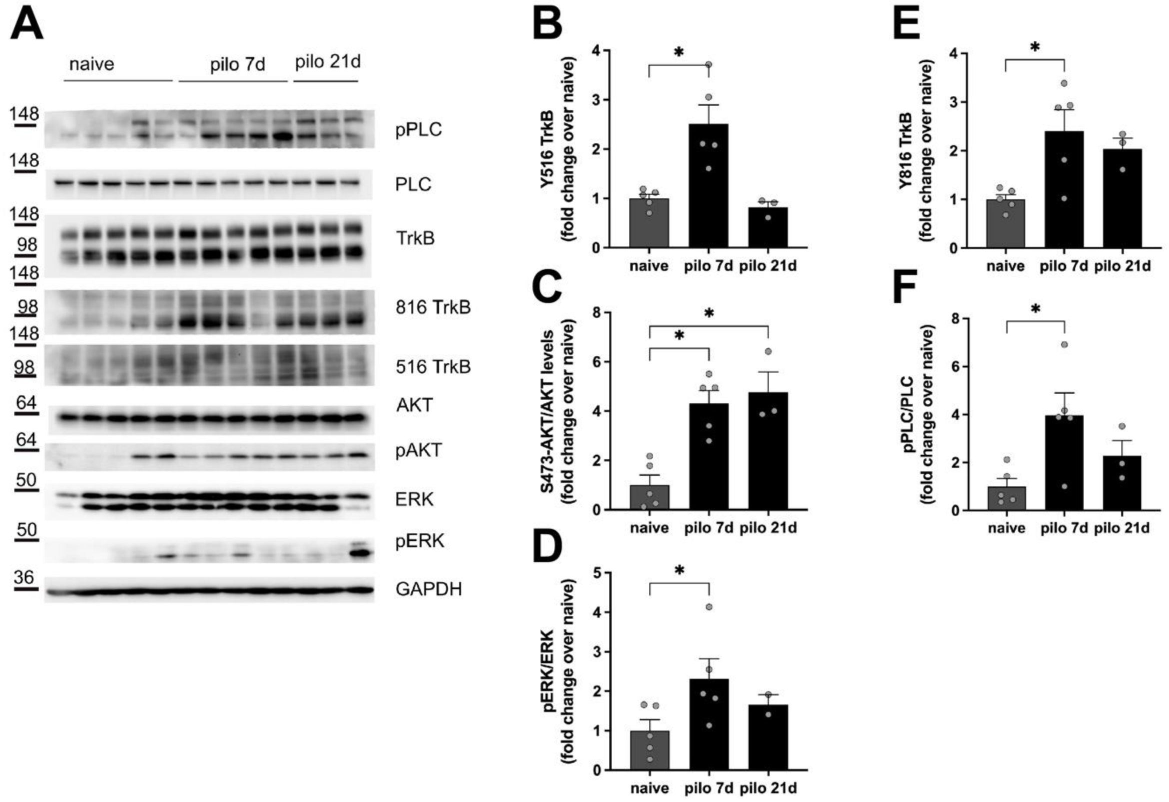

Supplementary Figure 2. Phosphorylation of TrkB, AKT, ERK and PLCγ proteins in the hippocampi of lithium-pilocarpine (pilo) rats. (**A**) Representative western blot of the indicated proteins in extracts from hippocampi. (**B-F**) Quantification of Y515 TrkB (B), S473-AKT (C), ERK (D), Y816 TrkB (E) and Y783-PLCγ (F) phosphorylation. Protein levels are shown as fold change over naïve (vehicle-treated rats). Levels of phosphorylated proteins are normalized against the corresponding total protein, then for loading (GAPDH). Naïve: n=5; pilo 7 days: n=5; pilo 21 days; n=3. * p<0.05, Kruskal-Wallis one-way ANOVA and *post-hoc* Dunn's test.
